# Copper-decorated carbon nanotubes-based composite electrodes for nonenzymatic detection of glucose

**DOI:** 10.1186/1556-276X-7-266

**Published:** 2012-05-22

**Authors:** Aniela Pop, Florica Manea, Corina Orha, Sorina Motoc, Elida Ilinoiu, Nicolae Vaszilcsin, Joop Schoonman

**Affiliations:** 1 “Politehnica” University of Timisoara, Timisoara, 300006, Romania; 2 National Institute for Research and Development in Microtechnologies, Bucharest, 077190, Romania; 3 National Institute for Research and Development in Electrochemistry and Condensed Matter, Timisoara, 300569, Romania; 4 Department ChemE, Delft University of Technology, Delft, 2600, The Netherlands

**Keywords:** Multiwall carbon nanotubes composite electrodes, Copper particles, Glucose, Electrochemical determination

## Abstract

The aim of this study was to prepare three types of multiwall carbon nanotubes (CNT)-based composite electrodes and to modify their surface by copper electrodeposition for nonenzymatic oxidation and determination of glucose from aqueous solution. Copper-decorated multiwall carbon nanotubes composite electrode (Cu/CNT-epoxy) exhibited the highest sensitivity to glucose determination.

## Background

Nowadays, a large community of researchers is focusing on the development of different applications for carbon nanotubes (CNT) in the field of electrochemical sensing. The high sensitivity, chemical stability, excellent electrical conductivity, and modifiable surface that provides the possibility to fabricate multifunctional electrochemical sensors are only a few important properties which recommend them for sensing applications [1,2]. Using CNT in composites gives also the possibility to fabricate sensors with high electroanalytical performances with easy renewable surface and also a very good mechanical strength [1-3]. Nonenzymatic glucose determination in medical applications by electrocatalytic oxidation is of great interest to electrochemists [4-9]. Fabrication of high performance sensors for glucose continues to be a provocative challenge [7-10]. In this paper, three types of multiwall carbon nanotubes-based composite electrodes, i.e., CNT within an epoxy matrix (CNT-epoxy), CNT-synthetic A-type zeolite (SZ) within an epoxy matrix (SZCNT-epoxy), and CNT-natural clinoptilolite zeolite (NZ)-epoxy matrix (NZCNT-epoxy) were prepared, decorated with copper particles by electrodeposition, and then characterized and tested for direct electrochemical detection of glucose.

## Methods

Multiwall CNT with an average diameter of 9.5 nm and average length of 1.5 μm were purchased from Nanocyl, Belgium (Belgium Nanocyl, Sambreville, Belgium). A synthetic A-type zeolite (SZ) was prepared using natural clinoptilolite as a silicon source and sodium aluminate as aluminum source, as we previously described [11]. The composite electrodes were prepared by dispersion of CNT in tetrahydrofuran, 99.9% (THF, Sigma-Aldrich Laborchemikalien GmbH, Seelze, Germany) and epoxy resin (Araldite®LY5052, Huntsman Advanced Materials, Klybeckstrasse, Basle, Switzerland) by ultrasonication, followed by the homogenization of the resulting paste with the zeolite particles and the hardener using a two-roll mill. The mixture was then poured into polyvinyl chloride tubes and cured at 60°C for 24 h, obtaining disk electrodes with the surface area of 0.196 cm^2^. The ratios were chosen to reach 20% wt CNT, 20% wt SZ, and 20% wt NZ. The surface of prepared electrodes was then decorated with copper by electrodeposition at a potential of −0.5 V vs. Ag/AgCl for 20 s in the presence of 0.1 M CuSO_4_ solution. Scanning electron microscopy (SEM) was performed using an Inspect S PANalytical instrument (PANalytical Spectris Australia Pty Ltd., Sydney, New South Wales, Australia) coupled with the energy-dispersive X-ray analysis detector (EDX). Voltammetric and amperometric measurements were carried out using an Autolab PGSTAT101 (Metrohm Autolab, Utrecht, The Netherlands) controlled with NOVA 1.6 software (Metrohm Autolab, Utrecht, The Netherlands) and a three-electrode cell, with an Ag/AgCl reference electrode, a platinum counter electrode, and composite working electrodes.

## Results and discussion

### Surface characterization

**Figure 1:**
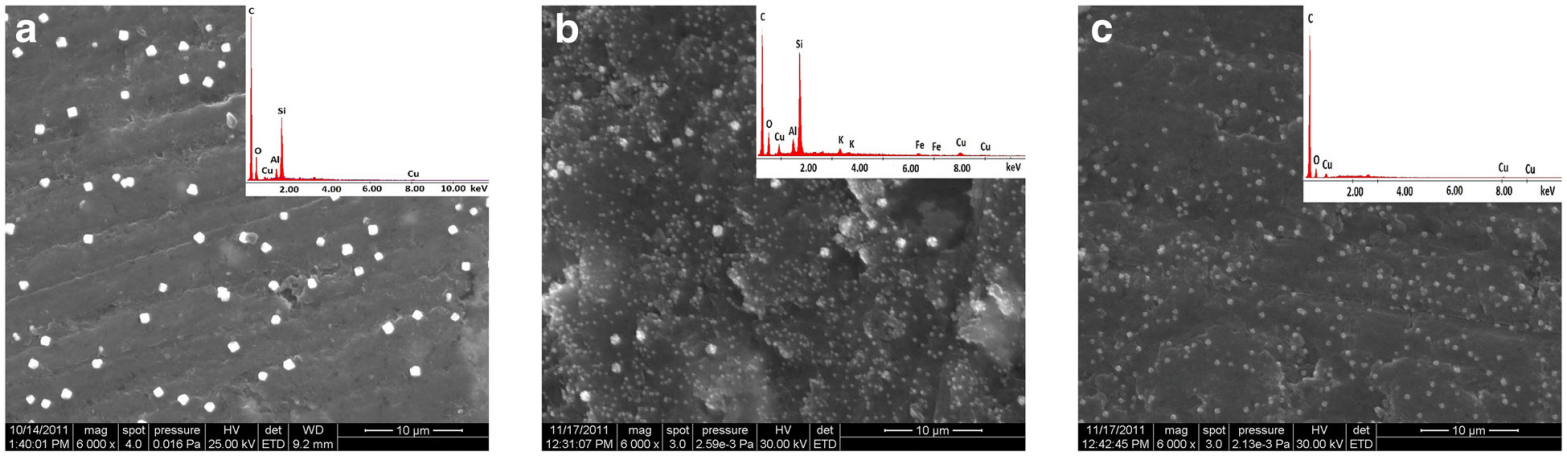
**SEM images and EDX quantification (Inset) of Cu.** SEM images and EDX quantification of Cu decorated (**a**) SZCNT-epoxy, (**b**) NZCNT-epoxy, and (**c**) CNT-epoxy electrode materials.

Figure 1 shows the SEM image of tested electrodes, i.e., SZCNT-epoxy, NZCNT-epoxy, and CNT-epoxy composite material decorated with Cu particles. The EDX data for the electrode composite material revealed the presence of copper on the composite electrode surface. The size of Cu particles depends on the electrode material. For the CNT-epoxy electrode, the Cu particles were distributed uniformly on the electrode surface and the particles size was around 300 nm. The nature and size of the zeolite influenced the electrodeposition process. Synthetic zeolite allowed reaching a better distribution of Cu particles in comparison with natural zeolite.

### Direct electrochemical detection of glucose

**Figure 2:**
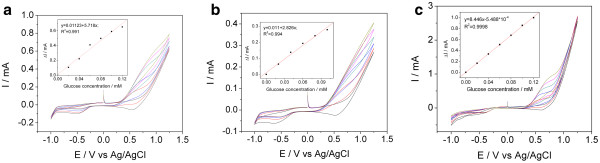
**Cyclic voltammograms.** Cyclic voltammograms of (**a**) Cu/SZCNT-epoxy, (**b**) Cu/NZCNT-epoxy, (**c**) Cu/CNT-Epoxy electrodes recorded in 0.1 M NaOH supporting electrolyte and after five successive additions of 0.02 mM glucose; potential scan rate 0.05 Vs^−1^. Insets: calibration plots of peak currents vs. glucose concentrations. Black lines, CV recorded in 0.1 M NaOH supporting electrolyte; red lines, CV recorded in 0.1 M NaOH supporting electrolyte and 0.02 mM glucose; blue lines, CV recorded in 0.1 M NaOH supporting electrolyte and 0.04 mM glucose; green lines, CV recorded in 0.1 M NaOH supporting electrolyte and 0.06 mM glucose; pink lines, CV recorded in 0.1 M NaOH supporting electrolyte and 0.08 mM glucose; and yellow green lines, CV recorded in 0.1 M NaOH supporting electrolyte and 0.1 mM glucose.

A series of cyclic voltammograms recorded at the tested Cu decorated electrodes in 0.1 M NaOH and in the concentration range 0.02 to 0.12 mM glucose is illustrated in Figure 2. All the electrodes exhibited a single oxidation peak in the potential range from +0.63 to +1.0 V vs. Ag/AgCl, and the glucose seems to be irreversibly oxidized at the tested composite electrodes. The best performances for glucose oxidation were noticed on the Cu/CNT-epoxy composite electrode due to the presence of higher content of copper particles with better electocatalytic properties towards glucose oxidation. The tested electrodes presented very high sensitivities for glucose, i.e., 8.446 mA∙mM^−1^ for Cu/CNT-epoxy, 5.718 mA∙mM^−1^ for Cu/SZCNT-epoxy and 2.826 mA∙mM^−1^ for Cu/NZCNT-epoxy, superior to the other values previous reported for catalyst-modified composite electrodes [7].

**Figure 3:**
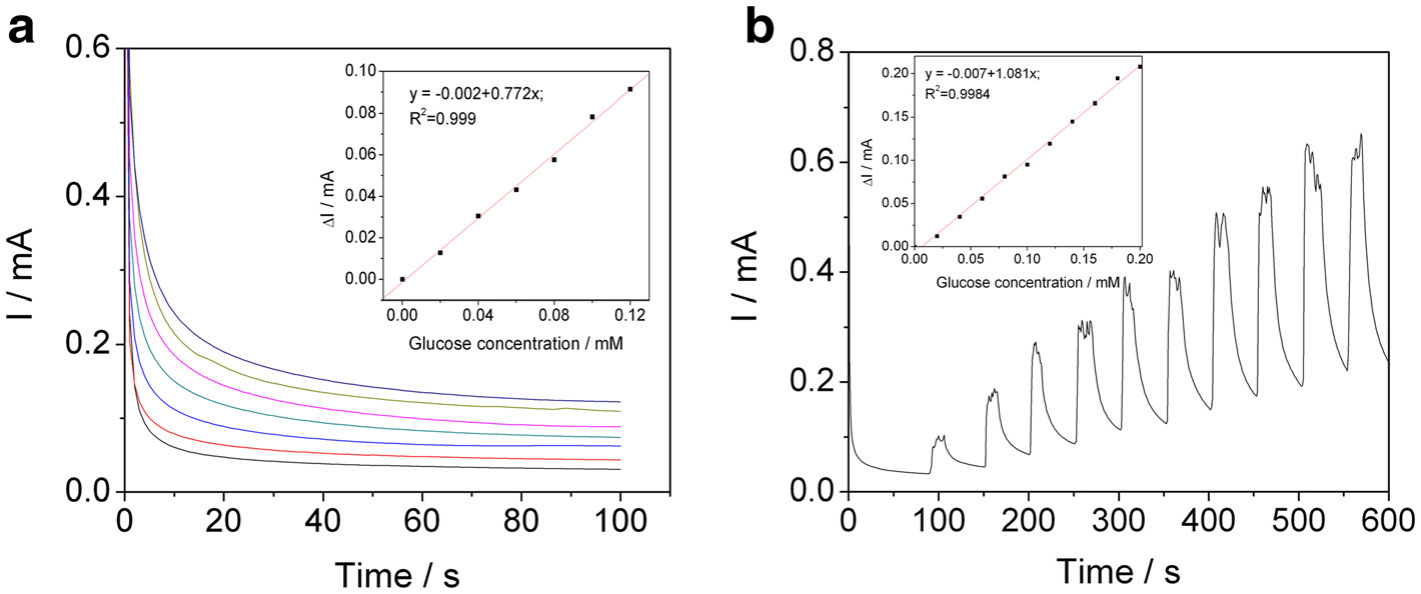
**Chronoamperometric response of Cu/CNT-Epoxy electrode recorded in 0.1 M NaOH supporting electrolyte.** Chronoamperometric response of Cu/CNT-Epoxy electrode recorded in 0.1 M NaOH supporting electrolyte and by successive additions of 0.02 mM glucose without (**a**) and under stirring condition (**b**). Insets: calibration plot of peak current vs. glucose concentration. Lines in (a): black lines, CV recorded in 0.1 M NaOH supporting electrolyte; red lines, CV recorded in 0.1 M NaOH supporting electrolyte and 0.02 mM glucose; blue lines, CV recorded in 0.1 M NaOH supporting electrolyte and 0.04 mM glucose; green lines, CV recorded in 0.1 M NaOH supporting electrolyte and 0.06 mM glucose; pink lines, CV recorded in 0.1 M NaOH supporting electrolyte and 0.08 mM glucose; and yellow green lines, CV recorded in 0.1 M NaOH supporting electrolyte and 0.1 mM glucose.

Further amperometric tests were performed using the best performing electrode for the determination of glucose selected by cyclic voltammetry, i.e., Cu/CNT-epoxy. The chronoamperometry was employed in two variants, without and under stirring conditions (see Figure 3). Parameters for amperometric measurements were established based on cyclic voltammetric responses i.e., in stationary conditions, the applied potential was settled at +0.7 V vs. Ag/AgCl. No “catalytic saturation” occurred during the test performed, revealing a very good composite electrode surface control. By applying this chronoamperometric technique (Figure 3a) without stirring, the sensitivity of Cu/CNT-epoxy electrode was calculated to be 0.772 mA∙mM^−1^, smaller than the one recorded by voltammetric techniques.

**Figure 4:**
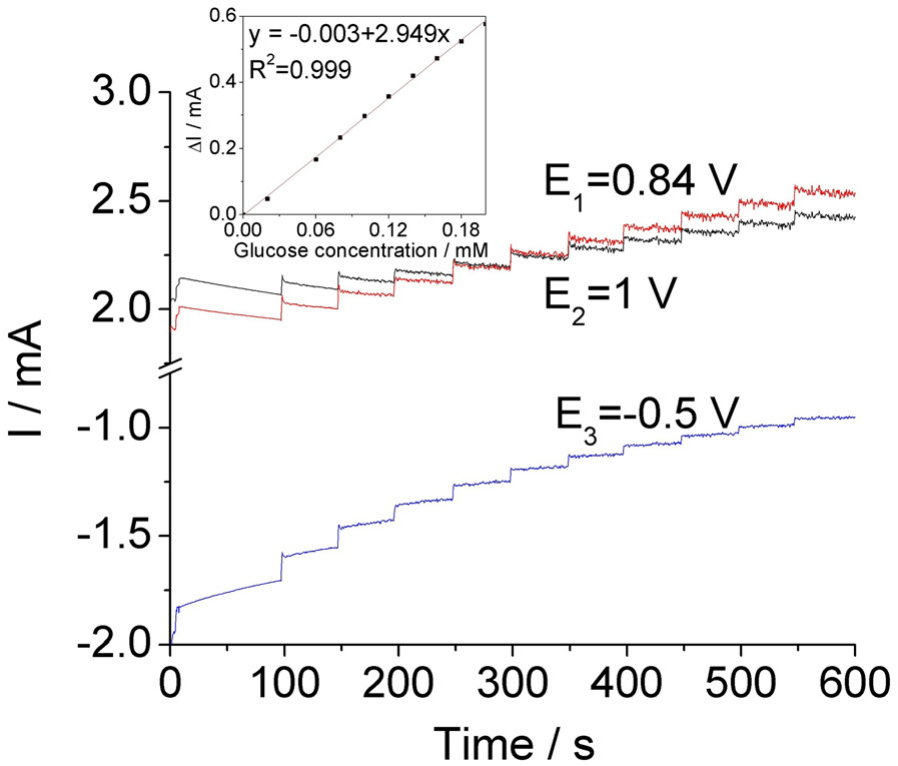
**Multiple-pulsed amperometric response of the Cu/CNT-epoxy electrode.** Multiple-pulsed amperometric response of the Cu/CNT-epoxy electrode for the successive and continuous addition of 0.02 mM glucose. Inset: calibration plot of peak current vs. glucose concentration.

Under the conditions of the batch system analysis, at an applied potential of +0.7 V vs. Ag/AgCl, the current response of glucose oxidation increased in straight line within the concentration range of 0.02 to 0.2 mM (Figure 3b). Due to the stirring conditions, this technique allowed to reach slightly better electroanalytical parameters, (i.e., sensitivity of 1.081 mA∙mM^−1^), in comparison with the amperometric measurements under stationary conditions. This fact reflects an oxidation process of glucose control by a rate-determining step involving mass transfer and a surface-controlled reaction. Better responses were reached under the batch system analysis conditions of multiple-pulsed amperometry (see Figure 4), operated at the optimal potentials, which were selected based on the potential range of glucose oxidation (see Figure 2). Thus, +0.84 V vs. Ag/AgCl was selected for detection, +0.50 V vs. Ag/AgCl for surface activation, and +1.0 V vs. Ag/AgCl as cleaning potential. A sensitivity of 2.949 mA∙mM^−1^ was reached by multiple-pulsed amperometry technique using the above-mentioned optimal potentials, which is the best of all applied amperometric techniques.

The reproducibility of the Cu/CNT-epoxy electrode using the multiple-pulsed amperometry was evaluated for three replicate measurements of glucose detection and relative standard deviations (RSD) of 3.5% shows a good reproducibility. A recovery test was also performed by analyzing three parallel glucose serum samples. This test was run for 0.1 mM glucose in 0.1 M NaOH supporting electrolyte, and a recovery of 98% was found with an RSD of 3.8% using multiple-pulsed amperometry.

## Conclusions

The electrocatalytic activities of the copper-decorated CNT-epoxy, SZCNT-epoxy and NZCNT-epoxy electrodes towards the oxidation of glucose in an alkaline solution were demonstrated. All copper-decorated composite electrodes exhibited useful properties for the direct oxidation and simple nonenzymatic determination of glucose on tested electrodes surface. The differences between the electroanalytical performances of the electrodes are related to composite structure and morphology, which influenced copper particle size and distribution on the surface of the composite material. The best electroanalytical performances obtained for the detection of glucose by cyclic voltammetry were recorded with the copper-decorated CNT-epoxy electrode, i.e., electrode sensitivity of 8.45 mA∙mM^-1^ and 0.2 μM glucose as limit of detection.

## Abbreviations

CNT, Carbon nanotubes; CNT-epoxy, Carbon nanotubes-epoxy electrode; Cu/CNT-epoxy, Carbon nanotubes-epoxy copper decorated electrode; Cu/NZCNT-epoxy, Carbon nanotubes-natural zeolite-epoxy copper decorated electrode; Cu/SZCNT-epoxy, Carbon nanotubes-synthetic zeolite-epoxy copper decorated electrode; EDX, Energy-dispersive X-ray spectroscopy; NZ, Natural zeolite; NZCNT-epoxy, Carbon nanotubes-natural zeolite-epoxy electrode; SEM, Scanning electron microscopy; SZ, Synthetic A-type zeolite; SZCNT-epoxy, Carbon nanotubes-synthetic zeolite-epoxy electrode.

## Competing interests

The authors declare that they have no competing interests.

## Authors’ contributions

AP carried out the electrodes preparation, electrochemical detection measurements and data interpretation. FM conceived the study and participated in data interpretation and coordination. CO performed SEM measurements and contributed at electrodes preparation. SM and EI contributed in the electrochemical characterization tests. NV and JS participated in paper design and coordination. All authors read and approved the final manuscript.

## Authors’ information

AP and CO are postdoctoral research associates. FM is an associate professor of Electrochemistry Applied for Environment Remediation and Monitoring. NV is a professor of Electrochemistry. JS is a professor of Advanced Materials. SM and EI are PhD students.
